# Anti-Cancer Roles of Probiotic-Derived P8 Protein in Colorectal Cancer Cell Line DLD-1

**DOI:** 10.3390/ijms24129857

**Published:** 2023-06-07

**Authors:** Byung Chull An, Jun Young Ahn, Daebeom Kwon, Sang Hee Kwak, Jin Young Heo, Seungwoo Kim, Yongku Ryu, Myung Jun Chung

**Affiliations:** R&D Center, Cell Biotech, Co., Ltd., 50 Aegibong-ro 409 Beon-gil, Gaegok-ri, Wolgot-myeon, Gimpo-si 10003, Gyeonggi-do, Republic of Korea; bcan@cellbiotech.com (B.C.A.);

**Keywords:** *Lactobacillus rhamnosus*, KCTC 12202BP, probiotics, P8, biotherapeutic, anti-cancer activity, anti-cancer target protein, protein-protein interaction

## Abstract

A novel probiotics-derived protein, P8, suppresses the growth of colorectal cancer (CRC). P8 can penetrate the cell membrane via endocytosis and cause cell cycle arrest in DLD-1 cells through down-regulation of CDK1/Cyclin B1. However, neither the protein involved in the endocytosis of P8 nor the cell cycle arrest targets of P8 are known. We identified two P8-interacting target proteins [importin subunit alpha-4 (KPNA3) and glycogen synthase kinase-3 beta (GSK3β)] using P8 as a bait in pull-down assays of DLD-1 cell lysates. Endocytosed P8 in the cytosol was found to bind specifically to GSK3β, preventing its inactivation by protein kinases AKT/CK1ε/PKA. The subsequent activation of GSK3β led to strong phosphorylation (S^33,37^/T^41^) of β-catenin, resulting in its subsequent degradation. P8 in the cytosol was also found to be translocated into the nucleus by KPNA3 and importin. In the nucleus, after its release, P8 binds directly to the intron regions of the *GSK3β* gene, leading to dysregulation of *GSK3β* transcription. GSK3β is a key protein kinase in Wnt signaling, which controls cell proliferation during CRC development. P8 can result in a cell cycle arrest morphology in CRC cells, even when they are in the Wnt ON signaling state.

## 1. Introduction

Colorectal cancer (CRC) is the third most common cancer and the third leading cause of cancer related deaths worldwide [1]. The International Agency for Research on Cancer (IARC) CancerBases has estimated more than 2.2 million individuals will be newly diagnosed with CRC annually by 2030 and that more than 1.1 million will die of this disease [2]. Treatment methods include surgery, radiation therapy, chemotherapy, targeted therapy and their combinations [3]. Chemotherapy agents include natural, synthetic, and biological substances to suppress or prevent CRC, but many of these agents are cytotoxic, killing normal (healthy) cells as well as tumor cells and inducing various types of adverse effects [4,5,6,7,8]. New drugs have thus been developed or are undergoing clinical trials to help improve current treatments for CRC patients.

Probiotics are beneficial microbes that inhabit human intestines. People have been taking probiotics for several centuries through various fermented foods, including yoghurt, cheese, and kimchi [9,10]. Additionally, probiotics have not been reported to be pathogens in any disease [11]. Due to their safety [12,13] and potency [14,15,16,17], probiotics may be utilized as drug delivery vehicles, including for the treatment of CRC [14,18].

Several probiotics have been shown to have anti-cancer activity, leading to the identification of proteins responsible for these properties [13,19,20,21]. Among these proteins is P8, which was isolated from the probiotic bacterium *Lactobacillus rhamnosus* (*L. rhamnosus*) and shown to have anti-cancer activity in the CRC cell line DLD-1. P8 was cloned into *Pediococcus pentosaceus* (*P. pentosaceus*), which was employed as a delivery vehicle for P8 expression and secretion in the intestines [14]. P8 has been shown to inhibit the growth of DLD-1 cells resulting in G_2_ cell cycle arrest through the reduction of CDK1/Cyclin B1 expression [14,15]. Further details about the anti-CRC mechanism of action of P8 have not been determined, including its initial target and the mechanism by which its anti-cancer signaling leads to cell cycle arrest.

The present study has sought to identify P8-interacting targets using P8 protein and DNA pull-down of DLD-1 cell lysates and gDNA fragments, respectively. Target proteins were identified with the LC-MS/MS method and target genes were identified using NGS analysis.

## 2. Results

### 2.1. Localization of P8 in DLD-1 Cells

**Figure 1:**
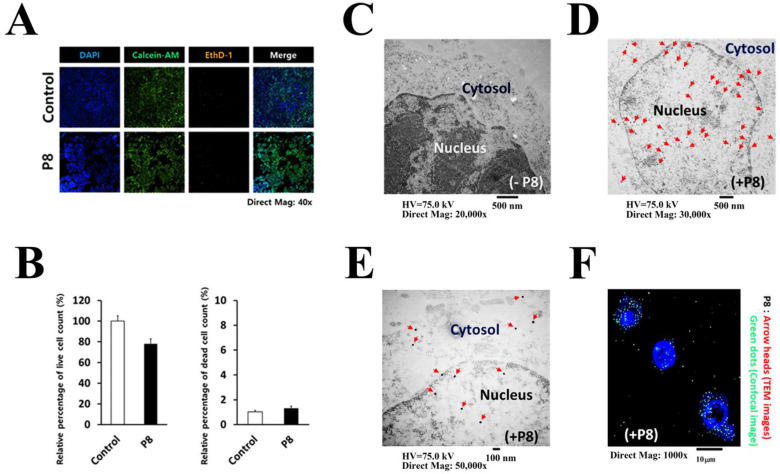
Anti-cancer activity of P8 and its action points in DLD-1 cells. (**A**) P8 significantly inhibits DLD-1 cell growth, whereas P8 derived a cell death is not observed. Anti-cancer efficacy of P8 was examined under an ImageXpress^®^Micro Confocal microscope system. DLD-1 cells treated with P8 for 72 h and then were stained with the live/dead cell markers Calcein-AM (Green)/EthD-1 (Red) or with the total cell marker DAPI (Blue). (**B**) Both live/dead cell amounts are represented using the average bar graphs. Results are presented as the average of three independent experiments conducted in duplicate. (**C**) Transmission electron microscopy (TEM) cross-section images of DLD-1 cells treated with P8 for 72 h. TEM was utilized to visualize DLD-1 cells. Negative control (without P8) (**D**,**E**). P8 treated DLD-1 cells (+P8). Red arrowheads indicate P8 which are widely spread in DLD-1 cells. Confocal microscopy cross-section image of DLD-1 cells treated with P8 for 72 h. (**F**) P8 treated DLD-1 cells (+P8). Green dots indicate P8 which are widely spread in DLD-1 cells. Confocal microscopy cross-section image of DLD-1 cells treated with P8 for 72 h. The nucleuses were stained with DAPI (blue).

The probiotic-derived P8 protein was previously shown to inhibit the proliferation of DLD-1 cells (Figure 1A,B) and to specifically down-regulate the cell cycle-related proteins CDK1 and Cyclin B1 [14,15]. Because cell cycle arrest is regulated in the nucleus, P8 has to penetrate the cell membrane and nuclear envelope to enter the nucleus. P8 was found to infiltrate into cells through endocytosis [15], with both transmission electron microscopy (TEM) (Figure 1C–E) and confocal microscopy (Figure 1F) showing that P8 proteins were present in both the cytosol and nucleus of DLD-1 cells.

### 2.2. Identification of P8 Targets in DLD-1 Cells

**Figure 2:**
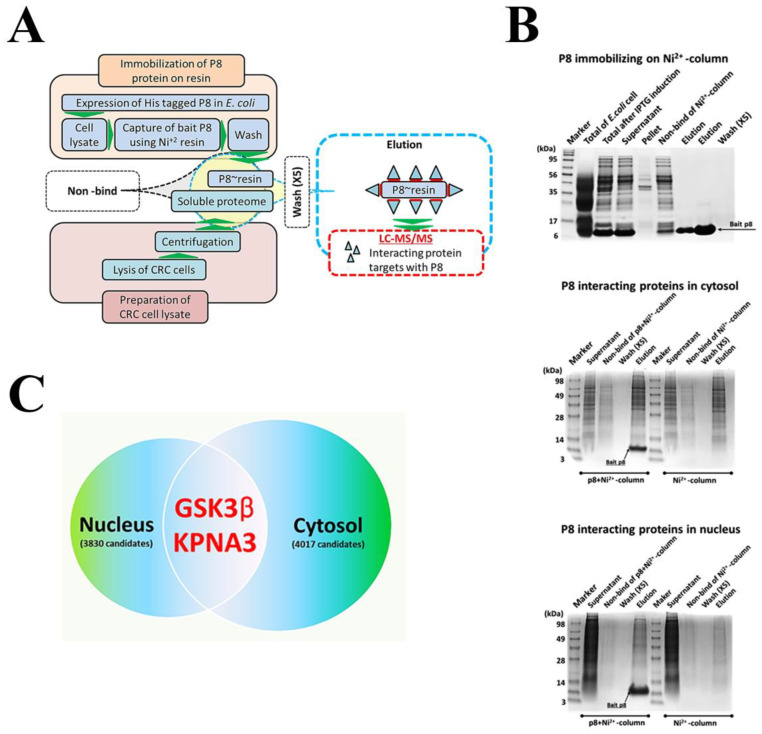
Profiling of target proteins interacting with P8. (**A**) Schematic diagram for profiling of proteins interacting with P8. Strategy developed for profiling P8-interacting target proteins from DLD-1 cell lysates using LC-MS/MS. (**B**) SDS-PAGE electrophoresis patterns for each step in the procedure for profiling of P8-interacting proteins using pull-down method. P8 immobilizing on Ni^2+^-column; capture of over-expressed P8 proteins with Ni^2+^ resin and purity of the P8 proteins immobilized on Ni^2+^ resin. P8-interacting proteins in cytosol; pull-down of P8-interacting proteins from DLD-1 cytosol fraction using P8-Ni^2+^ resin. P8-interacting proteins in nucleus; pull-down of P8-interacting proteins from DLD-1 nucleus fraction using P8-Ni^2+^ resin. Ni^2+^ resin (without P8) was used as a negative control at both pull-down experiments. (**C**) Selection of P8-interacting proteins common to lysates of DLD-1 cytosol and nucleus fractions. Consequently, Venn diagram showing overlap of P8 target proteins in the DLD-1 cell lysate fractions. Distribution of individual experiments of P8 target profiling in DLD-1 cell lysates. The numbers in parentheses indicate the numbers of detectable targets.

To understand the mechanism of action by which P8 inhibited DLD-1 proliferation, proteins interacting with P8 were identified in the cytosol and nucleus of P8 from DLD-1 cell lysates (Figure 2A). Cell lysates were incubated with His-tagged P8 bound to Ni^2+^-NTA resin or resin alone and the bound proteins eluted (Figure 2B). Differences were observed between proteins bound with resin alone and by resin bound to P8. Two proteins bound to P8 were identified as putatively interacting with P8, with systemic target profiling and LC-MS/MS suggesting that these proteins were involved in P8 transport and anti-proliferative activity (Figure 2C, Table 1).

### 2.3. Involvement of P8 Targets in P8 Infiltration and Activity

**Figure 3:**
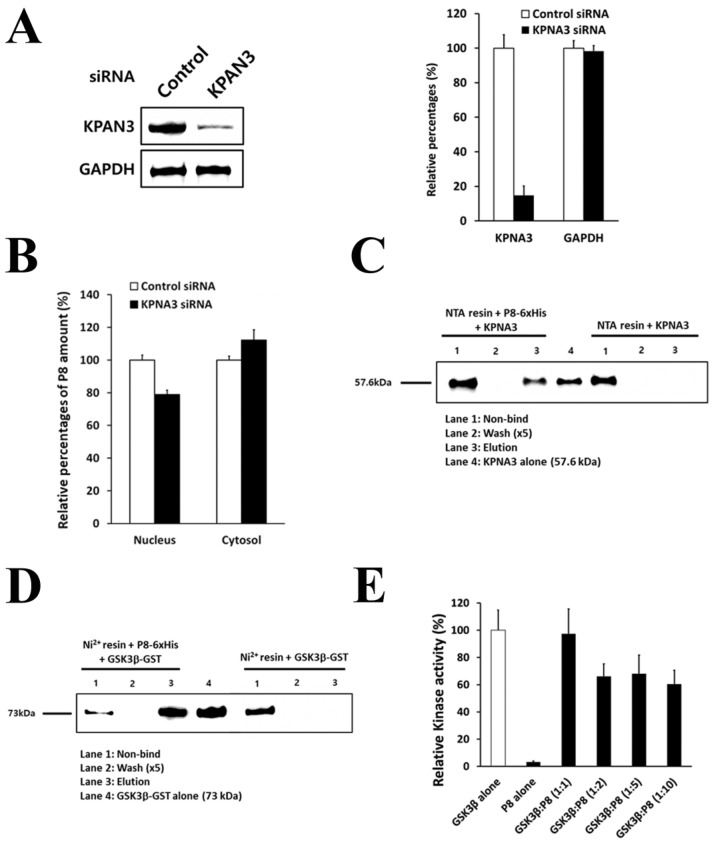
Identification of P8-interacting target proteins responsible for penetration and anti-proliferation. Identification of P8-interacting target protein responsible for penetration. (**A**) DLD-1 cells expressing endogenous P8 were treated with KPNA3 siRNA to knock out KPNA3. Effect of KPNA3 knockout on KPNA3 expression. Band intensities of the western blot result are calculated using Image J software (available online: https://imagej.nih.gov/ij/download.html, accessed on 31 May 2023). (**B**) Effects of KPNA3 siRNA on P8 expression in cell lysate fractions. P8 amount was calculated using the ELISA method. (**C**) Pull-down of KNPA3 with P8- Ni^2+^ resin. Results presented are the average of three independent experiments, each using duplicate samples. Identification of P8-interacting target proteins responsible for anti-proliferation. (**D**) Pull-down of GSK3β with P8+ Ni^2+^ resin. (**E**) Effects of molar ratios of GSK3β to P8 on GSK3β kinase activity. Results presented are the average of three independent experiments, each using duplicate samples.

One of the proteins identified with P8 pull-down assays was the nuclear transport receptor KPNA3 [22]. Knock-down of KPNA3 by transfection of KPNA3 siRNA into DLD-1::pCIneo-P8 cells, which express P8 [15], reduced the amount of KPNA3 in these cells, as shown with western blotting, with image analysis showing that KPNA3 siRNA reduced KPNA3 expression ~85% in DLD-1::pCIneo-P8 cells (Figure 3A). P8 specific enzyme-linked immunosorbent assay (ELISA) results showed that knock-down of KPNA3 reduced the amount of P8 in the nucleus by ~21%, while increasing the amount of P8 in the cytosol by ~12% (Figure 3B), strongly suggesting that P8 may be translocated into the nucleus with the importin-KPNA3 import system through the nuclear envelope. P8 bait pull-down assays showed that KPNA3 bound to P8 (Figure 3C), suggesting that KPNA3 specifically interacted with P8 and that P8 can pass through the nuclear envelope as a cargo protein of the importin-KPNA3 import system.

### 2.4. P8 Targets the Anti-Proliferation Protein GSK3β in DLD-1 Cells

Although P8 has been shown to induce cell cycle arrest at the G_2_/M phase in DLD-1 cells [14,15], its anti-proliferative mechanism of action remains unclear. The second protein identified with P8 pull-down assays was GSK3β (Figure 3C and Table 1), a key protein kinase in the Wnt/ GSK3β/β-catenin phosphorylation cascade associated with Wnt signaling that may be critical in G_2_/M regulation [23]. To determine whether GSK3β interacts directly with P8, recombinant GSK3β-GST was applied to resin bound to recombinant P8, with the result showing that GSK3β directly interacted with P8 (Figure 3D). To investigate the effect of this interaction on the kinase activity of GSK3β, GSK3β was incubated with P8 at molar ratios of 1:1 to 1:10, with the results showing that GSK3β kinase activity was significantly inhibited by ~40% when mixed with P8 at molar ratios > 1:2 (Figure 3E). Despite this effect of P8 on GSK3β kinase activity, the amount of P8 penetrating the nuclei of DLD-1 cells might not exceed this molar ratio; therefore, under normal conditions, P8 may be unable to directly inhibit GSK3β kinase activity.

**Figure 4:**
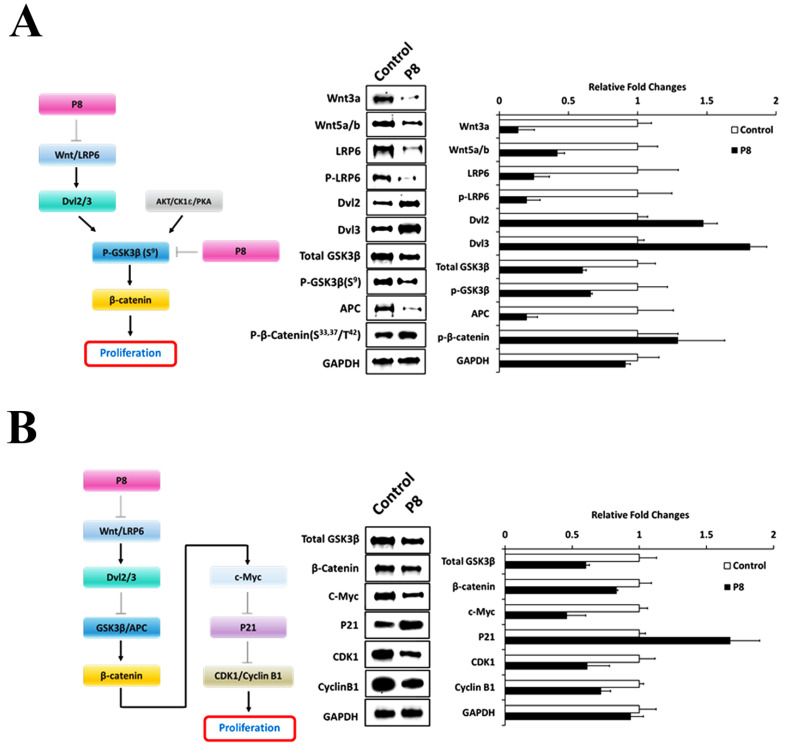
P8 is associated with anti-proliferative signaling. (**A**) Role of GSK3β as a key protein kinase in the Wnt signal pathway through a phosphorylation cascade resulting in β-catenin phosphorylation and subsequent degradation. P8 can specifically bind to GSK3β, significantly reducing its phosphorylation (S^9^) by AKT/CK1ε/PKA, and preserving the kinase activity of GSK3β and its phosphorylation of S^33,37^/T^41^ on β-catenin and its degradation by proteosomes. (**B**) P8 significantly reduced Wnt ligands using unknown mechanisms, reducing GSK3β and β-catenin and increasing the expression of P21, which suppresses CDK1 and Cyclin B1. Consequentially, P8 strongly promotes cell cycle arrest. Results presented are the average of three independent experiments, each with duplicate samples.

Analysis of the effects of P8 on the phosphorylation cascade in the Wnt/GSK3β/β-catenin signal pathway (Figure 4A) showed that P8 significantly reduced the phosphorylation of LRP6 (S^1490^) [24] and GSK3β (S^9^) [25] (Supplementary Figure S1), while increasing the phosphorylation of β-catenin (S^33,37^/T^41^) [26]. Phosphorylated β-catenin (S^33,37^/T^41^) was subsequently degraded in proteasomes after ubiquitination, resulting in cell arrest. This result suggested that P8 could strongly inhibit Wnt signaling in DLD-1 cells.

Analysis of the effects of P8 on the expression of proteins in the Wnt/GSK3β/β-catenin signal pathway (Figure 4A,B) showed that P8 markedly reduced the levels of almost all components of this pathway, including Wnt3a, Wnt5a/b, LRP6, GSK3β, APC, β-catenin, c-Myc, CDK1 and Cyclin B1 [27], while significantly increasing the expression of Dvl2, Dvl3 and P21. Overexpression of Dvls can lead to activation of the Wnt signal pathway independently of Wnt ligand stimulation [27,28], thereby suppressing GSK3β. Down-regulation of GSK3β and APC could reduce the levels of expression of β-catenin and c-Myc, inducing the expression of P21, which, in turn, suppresses the expression of G_2_ cell cycle factors, such as CDK1 and Cyclin B1 [14,15,17]. Taken together, these results strongly indicate that the Wnt signal pathway is a major target of P8 in DLD-1 cells.

### 2.5. P8 Down-Regulates the GSK3β Gene by Direct Interaction

**Figure 5:**
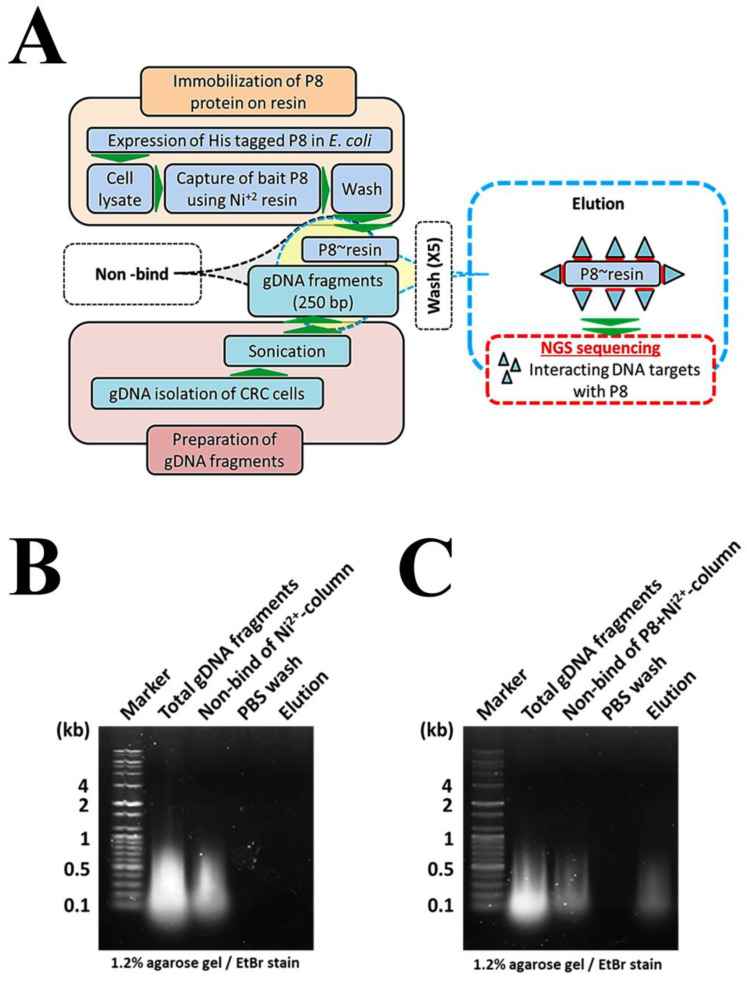
Profiling of P8-interacting target genes. (**A**) Schematic diagram for profiling genes targeted by P8. Strategy developed for profiling of P8-interacting target genes from DLD-1 gDNA fragments using the NGS sequencing method. (**B**,**C**) Agarose gel electrophoresis patterns for each step of the procedure used to profile P8-interacting target genes by DNA pull-down. Representative agarose gel electrophoresis results for each step in the procedure. P8 was immobilized on Ni^2+^ columns. (**B**) Negative control, consisting of Ni^2+^ column alone (without P8). No DNA bands were observed. (**C**) Pull-down of DLD-1 gDNA fragments directly interacting with P8 by P8+ Ni^2+^-columns. The sequences of the eluted DNA fragments were determined with NGS sequencing.

**Figure 6:**
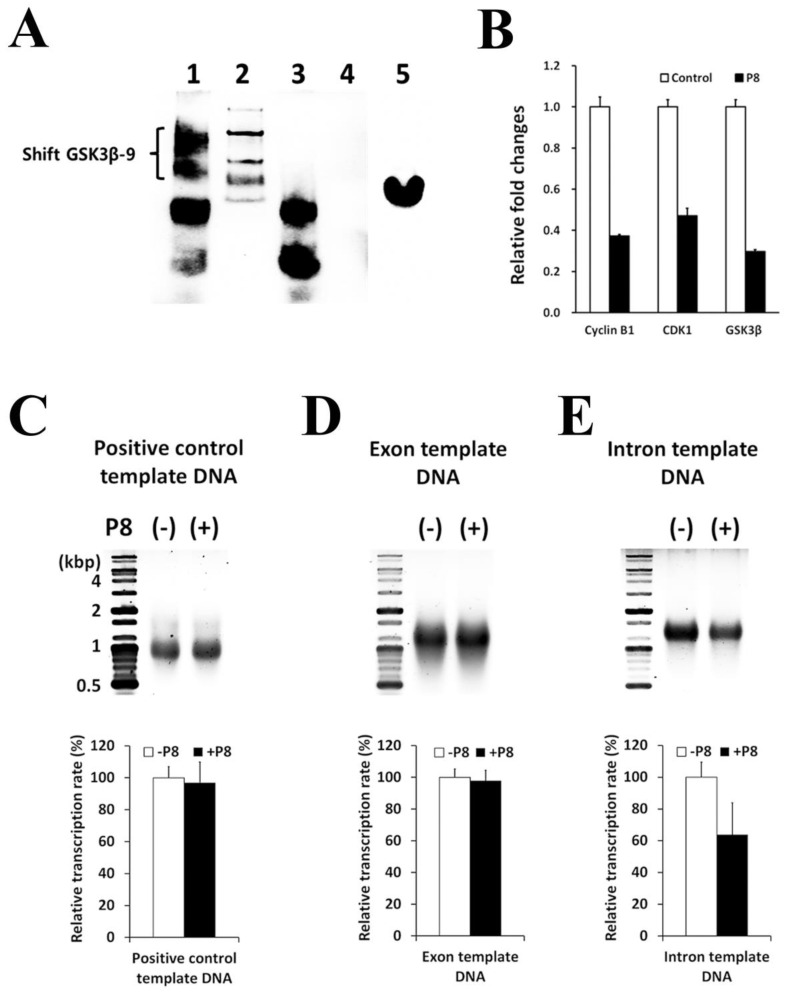
P8 down-regulates GSK3β by direct binding to the *GSK3β* gene. (**A**) Ability of P8 to bind to 17 sequences of the *GSK3β* gene (Table 2), as determined with EMSA. The 5′-biotin labeled GSK3β-9 showed a molecular weight shift in the presence (Lane 1), but not the absence (Lane 3) of P8, as visualized with avidin-HRP (Lane 4) wash fraction; (Lane 5) P8 alone visualized with rabbit anti-P8 IgG. (**B**) Effects of P8 on the levels of transcription of the genes encoding GSK3β, Cyclin B1 and CDK1, the latter two being indicators of cell arrest by P8. Results presented are the average of three independent experiments, each with duplicate samples. Effect of P8 on GSK3β gene transcription in vitro using GSK3β exon and intron template DNAs (Table 3). (**C**) Positive control from the in vitro transcription assay kit. (**D**,**E**) Effects of P8 on transcription from the GSK3β exon (**D**) and the GSK3β intron (**E**) template DNA. All transcription products were subjected to 1.2% agarose gel electrophoresis and ethidium bromide staining, followed with analysis of DNA band intensity using Image J software. Results presented are the average of three independent experiments, each with duplicate samples.

To investigate the mechanism by which P8 down-regulates the expression of the *GSK3β* gene in DLD-1 cells, P8 DNA pull-down assays were performed to isolate P8-interacting target genes (Figure 5A). Pulled-down genes were subjected to agarose gel electrophoresis (Figure 5B,C), followed by the elution of bound DNA fragments and their analysis by next generation sequencing (NGS). Seventeen sites on the *GSK3β* gene were found to interact with P8, with all of these targeted sites localized to introns (Table 2). These interactions were confirmed with electrophoretic mobility-shift assay (EMSA) using 17 5′-biotin labeled GSK3β EMSA probes synthesized by Cosmogenetech Co., Ltd. (Table 2). One of these probes, GSK3β-9, was significantly shifted to a higher molecular weight (MW) (Figure 6A); qPCR confirmed that P8 binding to the *GSK3β* gene reduced its level of transcription (Figure 6B). To further show that P8 interfered with *GSK3β* transcription, in vitro transcription assays were performed using three template DNAs (Table 3), an intron that included the GSK3β-9 probe, a *GSK3β* exon region and a positive control in the assay kit (Supplementary Figure S2). P8 only slightly reduced the transcription of the positive control and exon DNA templates (Figure 6C,D), whereas P8 specifically inhibited the transcription of the intron template DNA by ~40% (Figure 6E).

## 3. Discussion

Probiotic organisms may act as biopharmaceutical factories and have been screened for potential therapeutic agents [10,13,19]. Probiotics can be successfully engineered to secrete anti-CRC biopharmaceuticals [14,18]. One protein derived from probiotics, P8, was found to have anti-CRC therapeutic properties, inducing G_2_ cell cycle arrest [14,15,17]. However, the initial target of P8 and its anti-CRC signal pathway remain unclear.

The present study assessed the route of P8 penetration into cells and the molecular mechanisms associated with cell cycle arrest, including the mechanism by which P8 down-regulates GSK3β, its effect on GSK3β kinase activity in β-catenin regulation, and its role in the Wnt pathway. Although P8 was previously shown to enter the cytosol through endocytosis at the cell membrane, the mechanism by which it is translocated into the nucleus through the nuclear envelope had not been determined [15]. Additionally, P8 was found to arrest the cell cycle of CRC cells and to alter the levels of expression of cell cycle arrest markers, including P21, CDK1 and Cyclin B1, but not P53.

Using P8 pull-down assays of DLD-1 cell extracts, the present study found that P8 interacted directly with GSK3β. Components of the Wnt signal pathway, including GSK3β, play central roles in cancer development and homeostasis [29]. GSK3β has been considered a potential target for therapeutic intervention in cancer [30]. P8 was found to significantly suppress the phosphorylation of proteins in the Wnt signal pathway by inhibiting GSK3β (S^9^) phosphorylation by protein kinases, such as AKT, PKA and CK1ε, suggesting that P8 binds close to this site. GSK3β, in turn, can phosphorylate β-catenin (S^33,37^/T^41^), resulting in the proteasome-dependent degradation of the latter after its ubiquitination [31].

P8 also reduced the levels of expression of components of the Wnt signal pathway, leading to cell cycle arrest. P8 significantly reduced expression of the Wnt ligands 3a and 5a/b, as well as LRP6, but markedly increased the levels of Dvls 2 and 3. Wnt ligands 3a and 5a/b and LRP6 can act as initial triggers of CRC cell proliferation, whereas Dvls regulated β-catenin degradation through the cytoplasmic destruction of the AXIN/APC/GSK3β complex [31,32,33].

Suppression of CRC cell proliferation, as in the Wnt-off condition, requires the kinase activity of GSK3β in the Wnt pathway to be maintained. P8 significantly reduced the amounts of inactive GSK3β [phospho-GSK3β (S^9^)], enhancing CRC growth. The ability of P8 to inhibit phosphorylation on GSK3β (S^9^) may be associated with the direct protein–protein interactions between P8 and GSK3β.

GSK3β may also play a positive role in cancer cell proliferation, because a reduced expression of GSK3β was negatively associated with tumor cell survival and proliferation [34,35,36]. Additionally, aberrant overexpression of GSK3β has been observed in CRC patients, whereas the suppression of GSK3β expression had a negative effect on cancer proliferation [34,37]. The present study found that P8 significantly reduced the expression of both total GSK3β and β-catenin. These reductions may be associated with a decreased expression of c-Myc, which suppresses P21 expression [38,39]. Thus, a P8-associated increase in P21 expression would suppress the expression of CDK1 and Cyclin B1, both of which are G_2_ cell cycle markers [14,15,17].

Although the present study found that P8 interacted directly with GSK3β to suppress cell proliferation activity, as well as investigating the P8 derived anti-proliferation signal pathway, the mechanism by which P8 down-regulates GSK3β was still unclear. The ability of P8 to directly affect *GSK3β* transcription was therefore evaluated with P8 pull-down of gDNA fragments of DLD-1 cells. Because P8 was not homologous with any enzymes, P8 was thought to directly affect *GSK3β* transcription as a transcription factor. DNA fragments interacting with P8 were subjected to NGS sequencing, with 17 sequences on the *GSK3β* gene identified. EMSA showed that one of these sequences, GSK3β-9, was bound to P8, as shown by an increase in MW. The P8-associated reduction of *GSK3β* mRNA levels in DLD-1 cells was confirmed with qPCR, and the P8-associated direct inhibition of GSK3β transcription was confirmed with in vitro transcription assays.

**Figure 7:**
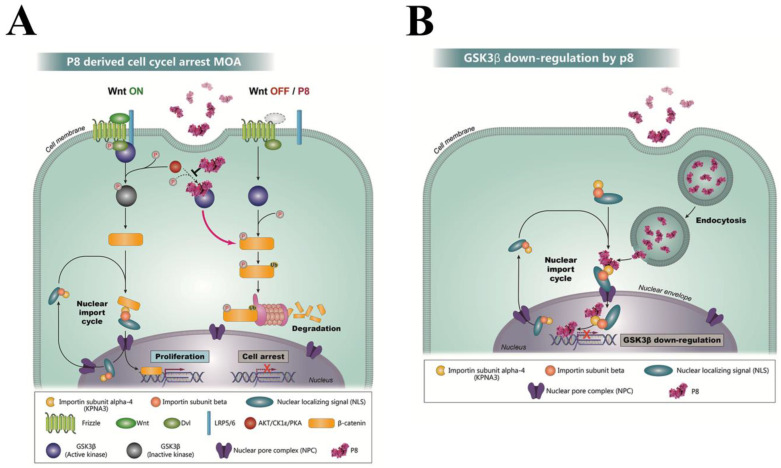
P8 has anti-proliferative activity in Wnt signaling. (**A**) Mechanism with which P8 enters DLD-1 cells and directly down-regulates the GSK3β gene. P8, which enters the cytosol through endocytosis, binds to KPNA3, a component of the importin transport system, at the nuclear envelope. The P8-KPNA3 complex is translocated into the nucleus through NPC. Following its release from the importin transport system, P8 binds to a specific intron sequence on the GSK3β gene, significantly inhibiting GSK3β gene transcription. (**B**) Wnt signaling in CRC is a major modulator of cell proliferation, self-renewal, differentiation, and tissue homeostasis during tumor development. Hyper-activation of Wnt signaling is observed in almost CRCs. When Wnt signaling is suppressed, GSK3β initiates a phosphorylation cascade, phosphorylating β-catenin and resulting in its subsequent degradation. By contrast, activation of Wnt signaling results in GSK3β inactivation by phosphorylation (S^9^), resulting in non-phosphorylation of β-catenin, with the latter remaining active and promoting cell proliferation. Binding of P8 to GSK3β inhibits the phosphorylation (S^9^) of GSK3β by the protein kinases AKT, CK1ε, and PKA. Active GSK3β, in turn, phosphorylates β-catenin, resulting in its degradation. Consequently, P8 can induce cell cycle arrest in CRC cells, even when Wnt signaling is activated.

Taken together, these results indicate that P8 acts to suppress the cell cycle through multiple pathways. P8 initially enters CRC cells through endocytosis and binds directly to GSK3β. The latter significantly inhibits the Wnt pathway and increases Dvl2/3, resulting in the preservation of GSK3β kinase activity. P8 also reduces the phosphorylation of GSK3β (S^9^), allowing the latter to maintain its kinase activity. Furthermore, reductions in the amounts of both GSK3β and β-catenin lead to a negative signal cascade during the CRC cell cycle, reducing both CDK1 and Cyclin B1 and resulting in G_2_ cell cycle arrest (Figure 7A).

Cytosolic P8 was found to be transported into the nucleus by binding to importin subunit alpha-4, with the released P8 in the nucleus binding directly to the *GSK3β* gene and down-regulating its transcription (Figure 7B).

## 4. Materials and Methods

### 4.1. Bacterial Strains and Culture

*Lactobacillus rhamnosus* (*L. rhamnosus*; KCTC 12202BP) was obtained from Cell Biotech Co., Ltd. (Gimpo-si, Republic of Korea), and *Escherichia coli* (*E. coli*) strains DH5α and C41 (DE3) were obtained from Novagen (Madison, WI, USA) [14].

### 4.2. Cell Culture

The human CRC cell line DLD-1 was obtained from the Korean Cell Line Bank (KCLB; Seoul, Republic of Korea). DLD-1 cells were maintained in Roswell Park Memorial Institute (RPMI)-1640 medium (Gibco, Grand Island, NY, USA) containing 10% fetal bovine serum (Gibco) and 1% penicillin/streptomycin (Gibco) at 37 °C in an atmosphere containing 5% CO_2_.

### 4.3. Purification of Recombinant P8 Protein from E. coli

The *P8* gene was synthesized for expression in *E. coli* cells with Cosmogenetech, Inc. (Seoul, Republic of Korea). The P8 DNA fragment was digested with *Nde*I/*EcoR*I (New England BioLabs, Ipswich, MA, USA) and cloned into the expression vector pET-28a (Promega, Madison, WI, USA). *E. coli* strain DH5α cells were transformed with this construct, and *E. coli* strain C41(DE3) cells (Novagen) were transformed with the pET28a::P8 construct. The cells were cultured in M9 medium until its optical density at 600 nm reached 0.6. Expression of P8, containing a hexa-histidine (6×His) tag and a TEV protease cleavage site at the N-terminus, was induced by culture for 4 h in a medium containing 0.5 mM isopropyl β-D-1-thiogalacto-pyranoside (IPTG). The P8-overexpressing cells were harvested and resuspended in a 20 mM HEPES (pH 7.5), 150 mM NaCl buffer. The cells were sonicated and centrifuged and P8 was purified using incubating the cell supernatants with Ni^2+^-NTA agarose (Qiagen, Valencia, CA, USA), followed by washing with 20 mM HEPES (pH 7.5), 150 mM NaCl, 20 mM imidazole, and elution with 20 mM HEPES (pH 7.5), 150 mM NaCl, 300 mM imidazole. The 6×His tag was removed using TEV protease in the presence of 1 mM DTT. The homogeneity of the purified P8 protein was assessed using a size exclusion column (HiLoad 26/60 Superdex 200 pg; GE Healthcare, NJ, USA) equilibrated with 20 mM HEPES (pH 7.5), 150 mM NaCl.

### 4.4. Profiling of Proteins from DLD-1 Lysates Interacting with P8

Proteins in DLD-1 lysates directly interacting with P8 were isolated as described in Figure 2A. Briefly, recombinant 6×His-tagged P8 protein (6×His-P8) over-expressed in *E. coli* C41(DE3) was bound to Ni^2+^-NTA resin in a binding buffer. Ni^2+^-NTA resin bound to 6×His-tagged P8 was washed three times with washing buffer and incubated with soluble DLD-1 cell lysate at 4 °C for 24 h. The resulting complexes of Ni^2+^-NTA resin, P8, and proteins interacting with P8 were washed five times with PBS and complexes of 6×His-tagged P8 bound to its interacting proteins eluted with elution buffer. Ni^2+^-NTA resin without His-tagged P8 protein was used as a negative control. The complexes of P8 and its interacting proteins were separated using SDS-PAGE gradient gel electrophoresis. Individual protein bands were visualized by staining with Coomassie Brilliant Blue, and protein-containing gel slices were digested with trypsin, analyzed using LC-MS/MS, and identified using the PS1 identification program (Proteinworks Co., Ltd.; Daejeon, Republic of Korea) [40,41,42,43].

### 4.5. Co-Pull-Down Assays

For immunoblotting, P8 and GSK3β proteins were incubated in PBS buffer at 4 °C for 1 day. A pre-incubated mixture of 6×His-P8 and GST-GSK3β was fully immobilized onto Ni^2+^-NTA resin by incubation for 12 h at 4 °C, and the resin was washed thoroughly with PBS to remove excess 6×His-P8 and GST-GSK3β. As a negative control, GST-GSK3β without 6×His-P8 was immobilized onto Ni^2+^-NTA resin by incubation for 12 h at 4 °C, and the resin was washed with PBS to remove excess GST-GSK3β. The samples were separated on SDS-PAGE and transferred to PVDF membranes, which were processed for immunoblotting.

### 4.6. NGS Sequencing (Microgen, Inc.; Seoul, Republic of Korea)

#### 4.6.1. Truseq DNA Nano

The integrity of the gDNA was determined using agarose gel electrophoresis, and gDNA was quantified using Quant-IT PicoGreen (Invitrogen, MA, USA). Sequencing libraries were prepared using a TruSeq DNA Nano Library Prep Kit (Illumina, Inc., San Diego, CA, USA), according to the manufacturer’s instructions. Briefly, 100 ng of gDNA were fragmented using adaptive focused acoustic technology (AFA; Covaris, MA, USA) and the fragmented DNA was end-repaired to create 5′-phosphorylated, blunt-ended dsDNA molecules. The DNA was size-selected using a bead-based method, followed by the addition of a single adenine (A) base, and ligation of the Truseq indexing adapters. The purified libraries were quantified using qPCR according to the qPCR Quantification Protocol Guide (KAPA Library Quantification kits for Illumina Sequencing platforms) and qualified using the Agilent Technologies 2200 TapeStation (Agilent Technologies, CA, USA), followed by paired-end (2 × 150 bp) sequencing by Macrogen using the Novaseq platform (Illumina).

#### 4.6.2. Generation of Raw Data

The Illumina Platform generates raw images and base calling through the integrated primary analysis software (RTA; real time analysis; available online: https://knowledge.illumina.com/software/general/software-general-reference_material-list/000002211 accessed on 7 April 2023). The BCL/cBCL (base calls) binary was converted to FASTQ using the illumine package bcl2fastq2-v2.20.0. The demultiplexing option (--barcode-mismatches) was set to a perfect match (value: 0).

#### 4.6.3. Analysis Method

##### Alignment

Paired-end sequences produced using the HiSeq Instrument were first mapped to the hg38 human reference genome using the mapping program BWA (0.7.17). Mapping results were generated in BAM format, without unordered sequences and alternate haplotypes. PCR duplicates were marked using the MarkDuplicates.jar from the Picard-tools (2.18.2) package, which requires reads to be sorted. Reads with identical starting positions were considered duplicates and reduced to a single read. BAM files were subsequently recalibrated using Base Quality Score Recalibration (BQSR) of GATK (4.0.5.1). BQSR is a process that uses machine learning to model the sequencing errors empirically and adjust the quality scores accordingly.

##### Annotations of SNPs and Small Indels

Based on the BAM file, each sample underwent variant genotyping with the Haplotype Caller of GATK (4.0.5.1). At this stage, candidate SNPs and short indels were identified at single nucleotide resolution. Variants were filtered with Variant Filtration of GATK (4.0.5.1), a tool designed for hard filtering of variant calls based on certain criteria. Records were hard-filtered by changing the value in the FILTER field to something other than PASS. Filtered records were preserved in the output unless their removal was requested in the command line. Filtered variants were annotated with SnpEff (4.3t) software. The final product was in VCF format. Results were further annotated using an inhouse program and SnpSift and additional databases, including dbSNP138,151, ESP6500, 1000 Genome Project Phase3, ClinVar, and dbNSFP (3.5c) information.

##### Mapping Read Count

The coverage for the genome was calculated using genomecov of Bedtools (2.25.0). In regions of mappable mean depths over 50× the mapped reads were checked using IGV.

### 4.7. EMSA Assay

EMSAs were performed using the process described in [44], with PCR performed using the GSK3β-intron #9 DNA probes 5′-biotin-ATTTCTCAGCCAGCCGACACTCATGGAAAA-3′ (forward) and 5′-TTTCCATGAGTGTCGGCTGGCTGAGAAAT-3′ (reverse). The P8 protein and reaction buffer (PBS containing 2 mg of sonicated salmon sperm DNA) were added to a final volume of 20 µl. After incubation at 4 °C for 1 day, the mixtures of DNA probes and recombinant P8 protein were electrophoresed on native 8% acrylamide gel in 1× TGE buffer (50 mM Tris-Cl, pH 8.5, 1.9 mM glycine, 10 mM EDTA). Changes in the positions of DNA probes were visualized using avidin-HRP.

### 4.8. In Vitro GSK3β Kinase Assay

GSK3β kinase activity was measured using ADP-Glo™ Kinase Assay kits for GSK3β kinase (Promega), with activity determined by measuring the levels of ADP resulting from enzymatic phospho-transfer. To evaluate the effects of P8 on GSK3β kinase activity, luminescence was measured with or without P8 using a GloMax^®^ 96 microplate luminometer (Promega) according to the manufacturer’s instructions [45]. The effects of P8 concentration on GSK3β activity were determined by incubating 70 nM GSK3β with 70, 140, and 280 nM P8.

### 4.9. In Vitro Transcription Assay

In vitro transcription of an intron (target) and an exon (negative control) sequence of the *GSK3β* gene was assessed using EZ™ High Yield In Vitro Transcription kits (Enzynomics Co., Ltd., Daejeon, Republic of Korea). Conventional nonradioactive transcription assays were performed as described by the manufacturer. The DNA intron and exon templates in Table 3 were generated as described in Supplementary Figure S2. Briefly, the intron or exon sequence of *GSK3β* was inserted between the T7-promoter and -terminator of the pET28a vector, with the mature forms of these DNA templates prepared after restriction enzyme digestion (*Sph*I/*Pvu*I). RNA was synthesized from 1 ng of DNA template in the absence or presence of P8 protein (480 ng). The reaction products were subjected to 1% agarose gel electrophoresis, and relative transcriptional activity was analyzed using Image J software [46].

### 4.10. Western Blot Analysis

DLD-1 cells were lysed in RIPA lysis buffer containing a protease inhibitor cocktail (Roche). Aliquots containing 40 μg of protein were separated using SDS-PAGE and transferred to PVDF membranes (Amersham Bioscience, Piscataway, NJ, USA). The membranes were blocked by incubation with 5% skimmed milk in T-TBS and incubated overnight at 4 °C with appropriate primary antibodies (Cell Signaling Technology, Danvers, MA, USA); all diluted to 1:1000. The membranes were washed with T-TBS three times each for 15 min, again blocked in 5% skimmed milk/T-TBS, and incubated with an HRP-linked secondary antibody (Cell Signaling Technology, MA, USA) for 1 h at 4 °C. Glyceraldehyde 3-phosphate dehydrogenase (GAPDH) was used as an internal control. Protein bands were detected using an enhanced chemiluminescence kit (Millipore, Billerica, MA, USA), followed with autoradiography using a Chemi-doc™ Touch Imaging System (Bio-Rad Laboratories, Hercules, CA, USA) [14,15]. Band intensities of western blot results were calculated using Image J software.

### 4.11. Immunocytochemistry Using ImageXpress^®^ Micro Confocal Microscopy

DLD-1 cells were seeded onto coverslips placed in 6-well plates. After 24 h, P8 protein (0–40 μM) was added to each well for a further 72 h, and the cells were prepared for transmission electron microscopy (TEM) as described [47]. To reduce background signals, cells were blocked for 30 min with 4% bovine serum albumin in PBS and incubated overnight or for 2 h at 4 °C with a rabbit polyclonal anti-P8 antibody (Young In Frontier, Daejeon, Korea). Protein localization was visualized using FITC-conjugated goat anti-rabbit IgG (Jackson ImmunoResearch Laboratories, Inc.; West Grove, PA, USA). For nuclear staining, cells were incubated for 1 h at RT with 5 µg/mL Hoechst 33258 (Sigma, MO, USA), rinsed three times in PBS, and then mounted. Images were obtained under an ImageXpress^®^ Micro Confocal microscope (Molecular Devices, CA, USA).

### 4.12. Cell Proliferation Assay Using ImageXpress Live/Dead

DLD-1 cells were seeded in 96-well plates at a density of 1 × 10^3^ cells per well and incubated at 37 °C for 24 h. Various concentrations of recombinant P8 protein (0–40 μM) were added to each well, followed with incubation at 37 °C for an additional further 72 h. Cell viability was determined using the ImageXpress Live/Dead analysis module. Cells were subsequently stained with the Live/Dead cell markers Syto9 (Green) and EthD-1 (Red) or with the total cell marker Hoechst (Blue) [15].

## 5. Conclusions

P8 isolated from probiotics was shown to act as an anti-cancer biotherapeutic agent [15]. In previous studies, P8 has been shown to suppress the growth of CRC cells, inducing G_2_ cell cycle arrest by down-regulating CDK1/Cyclin B1 [14,15]. This present study identified the anti-cancer mechanisms of action of P8. First action, endocytosed P8 in the cytosol was found to be translocated into the nucleus by KPNA3 and importin, with nuclear P8 directly binding to GSK3β introns, disrupting its transcription. Second action, cytosolic P8 was also found to bind specifically to GSK3β, preventing its inactivation by protein kinases AKT/CK1ε/PKA. Active GSK3β was found to strongly phosphorylate β-catenin, resulting in its degradation. Consequently, P8 can induce cell cycle arrest in CRC cells, even when Wnt signaling is activated.

Finally, we have developed a novel probiotic-based drug delivery system using genetically engineered *P. pentosaceus*.SL4 (PP-P8) was found to successfully secrete P8 into human intestines [14,17]. Orally administered PP-P8 was found to be safe in rodent and marmoset models [12], suggesting its possible use in the treatment of patients with CRC. Moreover, PP-P8 has certain important advantages, including low production cost, safety, clear MOA, absence of toxicity, ability to be administered orally, and the ability to be combined with other treatment methods, including chemotherapy, radiation therapy, and targeted therapy.

## Figures and Tables

**Table 1:** Identification of p8 interacting target proteins by LC-MS/MS.

Target Proteins	Symbols	Accession # (UniProt)	Cellular Functions
**Glycogen synthase kinase-3 beta**	GSK3β	Q6FI27	Protein kinase activity
**Importin subunit alpha-4**	KPNA3	O00505	Nuclear protein import

**Table 2:** Profiling of p8 target sites on GSK3β gene.

GSK3B Targeting Sites of P8 Protein Based on NGS Sequencing Results		
Probe No.	Sites in 3 chr.	Probe Sequences (30 bp)
**GSK3** **β** **-1**	119,840,769	Sense: ATAAGGGGAACTTTAAAAAAAAGTATCTAT
**GSK3** **β** **-2**	119,861,831	Sense: ATGAAAATTGCCTAATAATACATTTCTCAG
**GSK3** **β** **-3**	119,867,698	Sense: GGTATTGAGAACAAAAAATGGCAGAACTCA
**GSK3** **β** **-4**	119,872,127	Sense: CTTATTAAAAATCCCTAATCAACCCTAACT
**GSK3** **β** **-5**	119,879,433	Sense: GATTTACCCACTTCAGCCTCCCAAAGTGTT
**GSK3** **β** **-6**	119,883,513	Sense: TTTCTGGAAAGGGCCAGACAGTAAATATT
**GSK3** **β** **-7**	119,888,927	Sense: TTTTCTGGAAAGGGCCAGACAGTAAATATT
**GSK3** **β** **-8**	119,889,190	Sense: CTTGCTGGTTTTGCAGCTCAGGTGGGCATC
*GSK3 **β** **-9**	119,889,294	Sense: ATTTCTCAGCCAGCCGACACTCATGGAAAA Anti-sense: TTTCCATGAGTGTCGGCTGGCTGAGAAAT
**GSK3** **β** **-10**	119,890,313	Sense: AGCATAAAAAGGAATAAACAGGTGATACAG
**GSK3** **β** **-11**	119,922,595	Sense: AATGGTTCTACTTTGATAACCCTTTTATTAT
**GSK3** **β** **-12**	119,955,234	Sense: AAAGAACCAACAGCTAAAAAAAAAAAAAAA
**GSK3** **β** **-13**	119,965,364	Sense: CCACCGTGCCCAGCCATTTTTTTTTTTTATT
**GSK3** **β** **-14**	119,981,533	Sense: GCACCCGCTGACAAGATGATTCTCTCCCGT
**GSK3** **β** **-15**	120,049,324	Sense: CTCCAGGCCTGGCCTGGGTGGTTTTAAAAT
**GSK3** **β** **-16**	120,059,642	Sense: TCTGAAATCTTAGTTCAACTTCCTCACCCA
**GSK3** **β** **-17**	120,085,808	Sense: ACATTCCGTCTTGAGAAAAAAAAAAAAGTAT

**Table 3:** Template DNA and primer sequences for In vitro transcription assay.

Names	Sequences
GSK3β-Intron template DNA	209627 gaca tatagttagg tgttttttaa ttgagtttga caatttctgc ctttgtaatt gaagtactta gactatttac attcagtgta attatcactg tggttgagtt taagttttgc accttcgtat ttgttttcct ttcatcccat ctttcctttg ctctgttttt ccccctcttt tactgccacc tatggattaa atcagtggtt tttatttttt ctattggctt ttaagctata cctccttgtt gcatttttag aggttggtct aggatttaaa atatgcatca atatattaca gtcagtcttc aagtagtaat gtaccatgtc atatagaaaa caagaaccgt gtgacagtat ttccattccc cttcctgttc tttgtgctat agttttcata ctttttaatt ctacatgtta taatccttac aatatttgtt gtttataggg ggaacccgcc cctaatattt caacataggt tttttctatt ttccatgagt gtcggctggc tgagaaataa agagaaagag tacaaagaga ggaattttac agctcggcct ccgggggtga catcacatat cagtagaacc gtgatgccca cctgagctgc aaaaccagca agttttatta aggatttcaa aaggggaggg gatgcaagaa cagggagtag gtcccaagat cacatgtttc atagggcaaa aggcagaaca aagatcacat gcttgtgagg aaacaggaca aaggacaaaa ggcagaactt ttgataaggg tctatgttct gcagtgcacg tattgtcttg ataaacatct taacagaaag cagggtttga gagcagagaa ctggtctgac ctcaaattta ccagggcggg atttttcccc accctgctaa gcctgagggt actgcaggag accagggcgg atttcagtcc ttatctctat ggcataagac agacactccc agagcagccg tttata 21056
GSK3β-Intron template DNA-F	CATATGGACATATAGTTAGG
GSK3β-Intron template DNA-R	GAATTCTATAAACGGCTGCT
GSK3β-Exon template DNA	275602 ccttccaca gttaagtttc agtgatacca tactcaggag tgggaagagg aaatcatatt cgtaatttca tttcgttgaa gccctgcctt tgttttggtt ctgaatgtct ttcctcctcg gtagcagtga gaccggtttc atttcatact tagtccattc agggacttag tgtagcacca gggagcccta gagctggagg atatcgaata gattaaattt tgctcgtctc ttccacaagc cctaaccatg ggtcttaaaa acagcagatt ctgggagcct tccatgctct ctctctctcc tcttttatct acttccctcc caaatgagag agtgacagag aattgttttt ttataaatcg aagtttctta atagtatcag gttttgatac gtcagtggtc taaaatgcta tagtgcaatt actagcagtt actgcacgga gtgccaccgt gccaatagag gactgttgtt ttaacaaggg aactcttagc ccatttcctc cctcccgcca tctctaccct tgctcaatga aatatcattt taatttcttt taaaaaaaat cagtttaatt cttactgtgt gcccaacacg aaggcctttt ttgaaagaaa aatagaatgt tttgcctcaa agtagtccatataaaatgtc ttgaatagaa gaaaaaacta ccaaaccaaa ggttactatt tttgaaacat cgtgtgttca ttccagcaag gcagaagact gcaccttctt tccagtgaca tgctgtgtca ttttttttaa gtcctcttaa tttttagaca catttttggt ttatgtttta acaatgtatg cctaaccagt catcttgtct gcaccaatgc aaaggtttct gagaggagta ttctctatccctgtggatat gaagacactg gcatttcatc tatttttccc tttccttttt aaaggattta actttggaat cttccaaagg aagtttggcc aatgccagat ccccaggaat ttgg 276594
GSK3β-Exon template DNA-F	CATATGCCTTCCACAGTTAA
GSK3β-Exon template DNA-R	GAATTCCCAAATTCCTGGGG
Amplification primer fair for pET28a::GSK3β-Intron/Exon template DNAs using PCR	
pET28a-F	GGAAGGGAAGAAAGCGAAAG
pET28a-R	GCAAGGAATGGTGCATGCAA

## Data Availability

The datasets used in current study are available from the corresponding author on reasonable request.

## Supplementary Materials

The supporting information can be downloaded at: https://www.mdpi.com/article/10.3390/ijms24129857/s1.

## Abbreviations

CRC	colorectal cancer
PP	*Pediococcus pentosaceus*
MOA	mode of action
KPNA3	importin subunit alpha-4
GSK3β	glycogen synthase kinase-3 beta
IARC	international agency for research on cancer
TEM	transmission electron microscopy
ELISA	enzyme-linked immunosorbent assay
NGS	next generation sequencing
EMSA	electrophoretic mobility-shift assay
MW	molecular weight
IPTG	β-D-1-thiogalacto-pyranoside
GAPDH	glyceraldehyde 3-phosphate dehydrogenase
BQSR	base quality score recalibration
MCS	multi cloning site.
