# Supplementary material for: Anti-Cancer Roles of Probiotic-Derived P8 Protein in Colorectal Cancer Cell Line DLD-1
Source: Int J Mol Sci. 2023 Jun 7;24(12):9857. doi: 10.3390/ijms24129857 (PMC10298382; doi:10.3390/ijms24129857)
Supplement: Supplementary file 1 [file ijms-24-09857-s001.zip › ijms-2360837-supplementary/Supplementary figure legends.pdf]

Supplementary figure legends

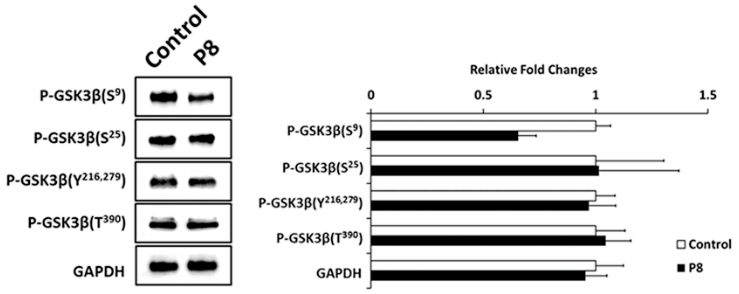

Supplementary Figure S1. Effects of P8 on phosphorylation of GSK3β. Effects of P8 on the phosphorylation of GSK3β using specific phospho-site antibodies. P8 significantly inhibited the GSK3β (S^9) phosphorylation by AKT/CK1ε/PKA, had no effects on phosphorylation at other sites. Results presented are the average of three independent experiments, each including duplicate samples.

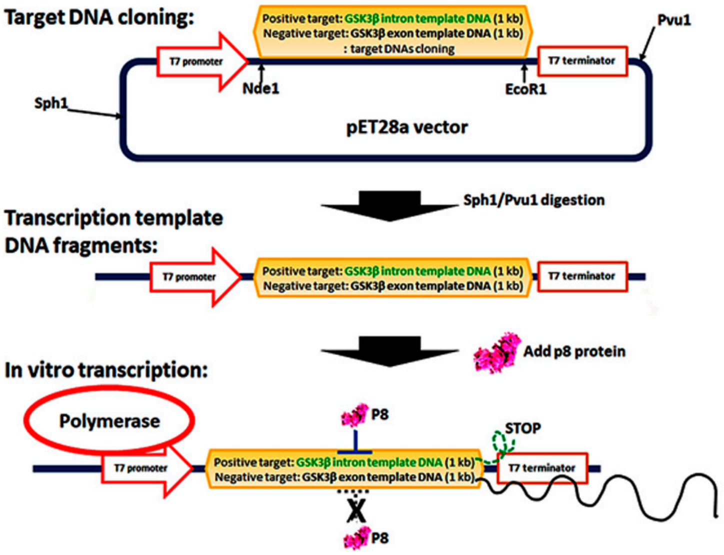

Supplementary Figure S2. Schematic diagram of the *in vitro* transcription assay. GSK3β template DNAs sited between T7 promoter and terminator were generated through three steps. During target DNA cloning, each ~1 kb template DNA fragment was PCR amplified and cloned into a multi cloning site (MCS) of the pET28a vector between T7 promoter and terminator. Each cloned sequence, including the T7 promoter

and terminator, was subsequently amplified by PCR. P8 protein was incubated with each template sequence to assess the effects of P8 on *in vitro* transcription.
