# Supplementary material for: Anti-Cancer Roles of Probiotic-Derived P8 Protein in Colorectal Cancer Cell Line DLD-1
Source: Int J Mol Sci. 2023 Jun 7;24(12):9857. doi: 10.3390/ijms24129857 (PMC10298382; doi:10.3390/ijms24129857)
Supplement: Supplementary file 1 [file ijms-24-09857-s001.zip › ijms-2360837-supplementary/Table S1. Identification of p8 interacting target proteins from DLD-1 cells.pdf]

Table S1. **Identification of p8 interacting target proteins by LC-MS/MS**

| Target proteins | Symbols | Accession # (UniProt) | Cellular functions |
|---|---|---|---|
| **Glycogen synthase kinase-3 beta** | GSK3β | Q6FI27 | Protein kinase activity |
| **Importin subunit alpha-4** | KPNA3 | O00505 | Nuclear protein import |

| Accession | Description | Coverage [%] | # Peptides | # PSMs | # Unique Peptides | # AAs | MW [kDa] | calc. pI | Score Sequest HT: Sequest HT |
|---|---|---|---|---|---|---|---|---|---|
| Q6FI27 | Glycogen synthase kinase-3 beta | 41 | 10 | 18 | 8 | 420 | 46.7 | 8.78 | 40.65 |

```
                     1          11         21         31         41         51         61         71         81         91
Modifications    1                C          O
Q6FI27             MSGRPRTTSF AESCKPVQQP SAFGSMKVSR DKDGSKVTTV VATPGQGPDR PQEVSYTDTK VIGNGSFGVV YQAKLCDSGE LVAIKKVLQD KRFKNRELQI

Modifications  101
Q6FI27             MRKLDHCNIV RLRYFFYSSG EKKDEVYLNL VLDYVPETVY RVARHYSRAK QTLPVIYVKL YMYQLFRSLA YIHSFGICHR DIKPQNLLLD PDTAVLKLCD

Modifications  201                                                                                         O
Q6FI27             FGSAKQLVRG EPNVSYICSR YYRAPELIFG ATDYTSSIDV WSAGCVLAEL LLGQPIFPGD SGVDQLVEII KVLGTPTREQ IREMNPNYTE FKFPQIKAHP

Modifications  301                 C                    C
Q6FI27             WTKVFRPRTP PEAIALCSRL LEYTPTARLT PLEACAHSFF DELRDPNVKL PNGRDTPALF NFTTQELSSN PPLATILIPP HARIQAAAST PTNATAASDA

Modifications  401
Q6FI27             NTGDRGQTNN AASASASNST
```

Coverage: 41.19%

Found Modifications:

C Carbamidomethyl (C)
O Oxidation (M)

| Accession | Description | Coverage [%] | # Peptides | # PSMs | # Unique Peptides | # AAs | MW [kDa] | calc. pI | Score Sequest HT: Sequest HT |
|---|---|---|---|---|---|---|---|---|---|
| O00505 | Importin subunit alpha-4 | 30 | 13 | 38 | 9 | 521 | 57.8 | 4.94 | 85.79 |

```
                     1          11         21         31         41         51         61         71         81         91
Modifications    1 M
O00505             MAENPSLENH RIKSFKNKGR DVETMRRHRN EVTVELRKNK RDEHLLKKRN VPQEESLEDS DVDADFKAQN VTLEAILQNA TSDNPVVQLS AVQAARKLLS

Modifications  101                                                                          C                    C
O00505             SDRNPPIDDL IKSGILPILV KCLERDDNPS LQFEAAWALT NIASGTSAQT QAVVQSNAVP LFLRLLRSPH QNVCEQAVWA LGNIIGDGPQ CRDYVISLGV

Modifications  201
O00505             VKPLLSFISP SIPITFLRNV TWVIVNLCRN KDPPPPMETV QEILPALCVL IYHTDINILV DTVWALSYLT DGGNEQIQMV IDSGVVPFLV PLLSHQEVKV

Modifications  301
O00505             QTAALRAVGN IVTGTDEQTQ VVLNCDVLSH FPNLLSHPKE KINKEAVWFL SNITAGNQQQ VQAVIDAGLI PMIIHQLAKG DFGTQKEAAW AISNLTISGR

Modifications  401                                                     C
O00505             KDQVEYLVQQ NVIPPFCNLL SVKDSQVVQV VLDGLKNILI MAGDEASTIA EIIEECGGLE KIEVLQQHEN EDIYKLAFEI IDQYFSGDDI DEDPCLIPEA

Modifications  501
O00505             TQGGTYNFDP TANLQTKEFN F
```

Coverage: 29.56%

Found Modifications:

C Carbamidomethyl (C)
M Met-loss+Acetyl (N-term)
