# Supplementary material for: Anti-Cancer Roles of Probiotic-Derived P8 Protein in Colorectal Cancer Cell Line DLD-1
Source: Int J Mol Sci. 2023 Jun 7;24(12):9857. doi: 10.3390/ijms24129857 (PMC10298382; doi:10.3390/ijms24129857)
Supplement: Supplementary file 1 [file ijms-24-09857-s001.zip › ijms-2360837-supplementary/Table S2. Profiling of p8 target sites on GSK3ÑΓ gene.pdf]

Table S2. Profiling of p8 target sites on GSK3β gene

GSK3β targeting sites of P8 protein based on NGS sequencing results		
Probe No.	Sites in 3 chr.	Probe sequences (30 bp)
**GSK3β-1**	119,840,769	Sense: ATAAGGGGAACTTTAAAAAAAAGTATCTAT
**GSK3β-2**	119,861,831	Sense: ATGAAAATTGCCTAATAATACATTTCTCAG
**GSK3β-3**	119,867,698	Sense: GGTATTGAGAACAAAAAATGGCAGAACTCA
**GSK3β-4**	119,872,127	Sense: CTTATTAAAAATCCCTAATCAACCCTAACT
**GSK3β-5**	119,879,433	Sense: GATTTACCCACTTCAGCCTCCCAAAGTGTT
**GSK3β-6**	119,883,513	Sense: TTTCTGGAAAGGGCCAGACAGTAAATATT
**GSK3β-7**	119,888,927	Sense: TTTTCTGGAAAGGGCCAGACAGTAAATATT
**GSK3β-8**	119,889,190	Sense: CTTGCTGGTTTTGCAGCTCAGGTGGGCATC
***GSK3β-9**	119,889,294	Sense: ATTTCTCAGCCAGCCGACACTCATGGAAAA
		Anti-sense: TTTCCATGAGTGTCGGCTGGCTGAGAAAT
**GSK3β-10**	119,890,313	Sense: AGCATAAAAAGGAATAAACAGGTGATACAG
**GSK3β-11**	119,922,595	Sense: AATGGTTCTACTTTGATAACCCTTTTATTAT
**GSK3β-12**	119,955,234	Sense: AAAGAACCAACAGCTAAAAAAAAAAAAAAA
**GSK3β-13**	119,965,364	Sense: CCACCGTGCCCAGCCATTTTTTTTTTTTATT
**GSK3β-14**	119,981,533	Sense: GCACCCGCTGACAAGATGATTCTCTCCCGT
**GSK3β-15**	120,049,324	Sense: CTCCAGGCCTGGCCTGGGTGGTTTTAAAAT
**GSK3β-16**	120,059,642	Sense: TCTGAAATCTTAGTTCAACTTCCTCACCCA
**GSK3β-17**	120,085,808	Sense: ACATTCCGTCTTGAGAAAAAAAAAAAAGTAT
