# Supplementary material for: Anti-Cancer Roles of Probiotic-Derived P8 Protein in Colorectal Cancer Cell Line DLD-1
Source: Int J Mol Sci. 2023 Jun 7;24(12):9857. doi: 10.3390/ijms24129857 (PMC10298382; doi:10.3390/ijms24129857)
Supplement: Supplementary file 1 [file ijms-24-09857-s001.zip › ijms-2360837-supplementary/Table S3. Template DNA and primer sequences for In vitro transcription assay.pdf]

Table S3. Template DNA and primer sequences for In vitro transcription assay

Names	Sequences
GSK3β-Intron template DNA	209627 gaca tatagttagg tgttttttaa ttgagtttga caatttctgc ctttgtaatt gaagtactta gactatttac attcagtgta attatcactg tggttgagtt taagttttgc accttcgtat ttgttttcct ttcatcccat ctttcctttg ctctgttttt ccccctcttt tactgccacc tatggattaa atcagtggtt tttatttttt ctattggctt ttaagctata cctccttgtt gcatttttag aggttggtct aggatttaaa atatgcatca atatattaca gtcagtcttc aagtagtaat gtaccatgtc atatagaaaa caagaaccgt gtgacagtat ttccattccc cttcctgttc tttgtgctat agttttcata ctttttaatt ctacatgtta taatccttac aatatttgtt gtttataggg ggaacccgcc cctaatattt caacataggt tttttctatt ttccatgagt gtcggctggc tgagaaataa agagaaagag tacaaagaga ggaattttac agctcggcct ccgggggtga catcacatat cagtagaacc gtgatgccca cctgagctgc aaaaccagca agttttatta aggatttcaa aaggggaggg gatgcaagaa cagggagtag gtcccaagat cacatgtttc atagggcaaa aggcagaaca aagatcacat gcttgtgagg aaacaggaca aaggacaaaa ggcagaactt ttgataaggg tctatgttct gcagtgcacg tattgtcttg ataaacatct taacagaaag cagggtttga gagcagagaa ctggtctgac ctcaaattta ccagggcggg atttttcccc accctgctaa gcctgagggt actgcaggag accagggcgg atttcagtcc ttatctctat ggcataagac agacactccc agagcagccg tttata 21056
GSK3β-Intron template DNA-F	CATATGGACATATAGTTAGG
GSK3β-Intron template DNA-R	GAATTCTATAAACGGCTGCT
GSK3β-Exon template DNA	275602 ccttccaca gttaagtttc agtgatacca tactcaggag tgggaagagg aaatcatatt cgtaatttca tttcgttgaa gccctgcctt tgttttggtt ctgaatgtct ttcctcctcg gtagcagtga gaccggtttc atttcatact tagtccattc agggacttag tgtagcacca gggagcccta gagctggagg atatcgaata gattaaattt tgctcgtctc ttccacaagc cctaaccatg ggtcttaaaa acagcagatt ctgggagcct tccatgctct ctctctctcc tcttttatct acttccctcc caaatgagag agtgacagag aattgttttt ttataaatcg aagtttctta atagtatcag gttttgatac gtcagtggtc taaaatgcta tagtgcaatt actagcagtt actgcacgga gtgccaccgt gccaatagag gactgttgtt ttaacaaggg aactcttagc ccatttcctc cctcccgcca tctctaccct tgctcaatga aatatcattt taatttcttt taaaaaaaat cagtttaatt cttactgtgt gcccaacacg aaggcctttt ttgaaagaaa aatagaatgt tttgcctcaa agtagtccatataaaatgtc ttgaatagaa gaaaaaacta ccaaaccaaa ggttactatt tttgaaacat cgtgtgttca ttccagcaag gcagaagact gcaccttctt tccagtgaca tgctgtgtca ttttttttaa gtcctcttaa tttttagaca catttttggt ttatgtttta acaatgtatg cctaaccagt catcttgtct gcaccaatgc aaaggtttct gagaggagta ttctctatccctgtggatat gaagacactg gcatttcatc tatttttccc tttccttttt aaaggattta actttggaat cttccaaagg aagtttggcc aatgccagat ccccaggaat ttgg 276594
GSK3β-Exon template DNA-F	CATATGCCTTCCACAGTTAA
GSK3β-Exon template DNA-R	GAATTCCCAAATTCCTGGGG
Amplification primer fair for pET28a::GSK3β-Intron/Exon template DNAs using PCR	
pET28a-F	GGAAGGGAAGAAAGCGAAAG
pET28a-R	GCAAGGAATGGTGCATGCAA
